# A Flexible Lockdown by Integrating Public Health and Economic Reactivation to Response the Crisis of COVID-19: Responses to Comments by Alvaro J Idrovo on “An Examination on the Transmission of COVID-19 and the Effect of Response Strategies: A Comparative Analysis”

**DOI:** 10.3390/ijerph17218068

**Published:** 2020-11-02

**Authors:** Yi-Tui Chen, Yung-Feng Yen, Shih-Heng Yu, Emily Chia-Yu Su

**Affiliations:** 1 Department of Health Care Management, College of Health Technology, National Taipei University of Nursing and Health Sciences, Taipei 10845, Taiwan; yitui@ntunhs.edu.tw (Y.-T.C.); dam37@tpech.gov.tw (Y.-F.Y.); 2 Section of Infectious Diseases, Taipei City Hospital, Yangming Branch, Taipei 11146, Taiwan; 3 Institute of Public Health, National Yang-Ming University, Taipei 11221, Taiwan; 4 Department of Business Management, National United University, Miaoli 36003, Taiwan; 5 Graduate Institute of Biomedical Informatics, College of Medical Science and Technology, Taipei Medical University, Taipei 11031, Taiwan; 6 Clinical Big Data Research Center, Taipei Medical University Hospital, Taipei 11031, Taiwan

## Abstract

We greatly appreciate Idrovo’s comments on our research and wish to specifically respond to his comments. Idrovo indicates that rapid increases in the number of confirmed cases in the past few weeks were observed in Latin America, where some countries had implemented stringent lockdowns for more than three months since the second half of March 2020. In his comments, Idrovo expresses his suspicion on the reality of lockdowns implemented in Latin America and worries about the negative impacts of lockdowns on economies. We thank the Editor for providing us with the opportunity to respond to Idrovo’s comments and explain parts of our study.

## Authors’ Response

According to the situation report released by the World Health Organization (WHO) [1], there have been 24,293,923 confirmed cases of COVID-19 in the world as of 28 August 2020. Currently, Latin America has become the most infected region from COVID-19 in the world. As of 28 August 2020, 3,717,156 confirmed cases have been recorded in Brazil, ranked the second highest in the world after the USA. Table 1 also shows that Peru, Mexico, Colombia, and Chile are in the top 10 countries with the most confirmed cases in the world. All of these countries are still fighting against the attack of COVID-19 currently.

Table 1 also reveals that new confirmed cases during the period of 17–23 August are still very high in these six countries. New confirmed cases in Brazil reached 256,810 cases, followed by Columbia (77,027 cases), Peru (59,771 cases), and so on. The proportion of new confirmed cases to cumulative confirmed cases (PNC) in Columbia and Argentina was 14.75% and 14.16%, respectively. The high PNC implies that the pandemic is still hitting and ravaging these countries. On 26 August 2020, Argentina recorded more than 10,000 new confirmed cases, reaching a new peak. Furthermore, the proportion of cumulative confirmed cases to population (PCP) in Chile was the highest on 23 August 2020, amounting to 20,700 cases per million population, while Mexico was the lowest with 4264 cases per million population.

Idrovo [2] emphasizes that many countries in Latin America have performed very stringent lockdown measures but the pandemic of COVID-19 is still roaring, and no sign appears to flatten the curve. Thus, he suggests that not all lockdowns in Latin America are real. However, several previous studies have shown that lockdown measures provide positive effects on the spread of virus [3,4]. Atangana [3] employs a mathematical model to examine the effect of lockdowns on the spread of COVID-19 and shows that lockdowns are effective to reduce the threat of COVID-19 in a few months. Atalan [4] examines the impact of the duration of lockdowns on the spread of COVID-19 by using data collected from 49 countries. The result of Atalan [4] finds that the duration of lockdowns is significantly correlated with the spread of the COVID pandemic, and thus, he suggests that COVID-19 transmission may be suppressed by lockdowns. The result in Chen et al. [5](2020) derived from the qualitative analysis emphasizes that the integration of viral tests with effective contact tracing systems, lockdowns, and public cooperation is vital for the successful mitigation of COVID-19 transmission. This result should be also appropriate to apply to Latin America. Thus, our response at first attempts to explain the gap between stringent lockdowns implemented in Latin America and the consequent result of ineffective mitigation of COVID-19 transmission.

Theoretically, lockdowns are a passive strategy to avoid the contact of the general public with the asymptomatically infected that are hidden in the public and have not been identified. In the absence of lockdowns, the rate of asymptomatic infection may increase proportionally to the social contacts of the infected. This means that the effectiveness of lockdowns to reduce the transmission of the infection depends on extensive viral tests together with effective contact tracing systems. Thus, effective response strategies should start from the identification of the infected hidden in the public through viral tests.

Extracted from Our World in Data [6], the number of daily viral tests and the number of positive rates for the six Latin American countries and the USA are listed in Table 2. All the Latin American countries have much less viral tests and higher positive rates than the USA. Mexico had tested just 8 people for every 100,000 population as of 21 August 2020 (the latest data available), ranking bottom among the six Latin American countries, and the positive rate (the share of tests returning a positive result) was 52.4%. The daily viral tests performed in other countries, except for Chile, were also very low, from 25 to 59 people per 100,000 population and positive rates stayed very high from 33.4% to 57.9% compared with the USA. The low number of daily viral tests together with high positive rates suggests that the viral testing implemented in these countries is not sufficient to identify all infected cases. Thus, several research works estimate that the number of confirmed cases may be underestimated in Latin America. For example, the number of infections in Brazil could be up to five times that of the official report due to the low levels of viral tests [7].

The number of viral tests and positive rates in Chile were 1.42 people per 1000 population and 6.5%, much better than other Latin American countries and only a little worse than the USA (2.24 people per 1000 population of daily tests and 5.8% positive rates). The higher daily tests and lower positive rates in Chile than other Latin American countries may be the major reason to explain the lower PNC of only 2.98% for Chile, indicated in Table 1. The low PNC in Chile implies that the control of COVID-19 transmission in Chile is better than other Latin American countries currently.

Based on the data of viral tests and positive rates indicated in Table 2, we suggest that insufficient viral tests are the major cause to explain the continual rising of new confirmed cases in some Latin American countries. Furthermore, contact tracing should be conducted to cut off the further transmission of COVID-19 from the infected to others after the infected person is identified. Our study [5] emphasizes that lack of an effective contact tracing system may lead to ineffective mitigation of COVID-19 transmission, even when the number of daily tests is high.

Without an accurate figure for the hidden asymptomatic infected and symptomatic cases and lacking an effective vaccine, a stringent lockdown becomes the most commonly used strategy to reduce COVID-19 transmission. However, a stringent lockdown measure in general has to restrict mobility across nation levels and change living patterns, including temporary closure of schools, retail and groceries, parks, transit stations, and residential areas. As a consequence, stringent lockdowns may depress the economy. Basically, a longer duration of lockdowns may cripple economies more when transportation systems, shopping centers, or factories are closed or restricted. The global gross domestic product growth would be reduced ranging from 3 to 6% [8] and each additional month of lockdown will reduce 2.5–3% of the global GDP. The baseline forecast published by the June 2020 Global Economic Prospects predicts a drop in global GDP by 5.2% for 2020 [9]. The forecast envisions a contraction of GDP by 7.2% in Latin America, 4.7% in Europe and Central Asia, 4.2% in the Middle East and North Africa, 2.7% in South Asia, while East Asia and the Pacific will grow by 0.5% only. Kanitkar [10] employs a linear input–output model to estimate the economic losses due to COVID-19 in India and finds GDP is probably reduced by 10–31%, depending on the duration of the lockdown. The results also indicate that power supply from coal-based power plants is reduced by 26%.

In consideration of the high crisis of economic depression due to COVID-19, lockdown measures should be taken to balance public health and economic consequences. Thus, we agree with Idrovo [2] that lockdowns may prevent the transmission of COVID-19 and a more flexible lockdown is required to reactivate a weak economy. A flexible lockdown is expected to simultaneously trade off both public health and economic restart, possibly to improve future life and to reopen the economy before the successful development of vaccines.

In the absence of previous experience, an approach of trial and error may be adopted to design a flexible lockdown. The key point to determine the intensity of lockdown measures depends on the control over the transmission and the demand for economic reactivation. In general, a flexible lockdown is imposed in a given area only or at the county-level. Mass gathering is still prohibited. Rapid testing kits are employed to obtain immediate results to identify the asymptomatic cases as early as possible. The application of high technology products like drones and unmanned transportation systems may be employed to reduce social meetings. For example, digital conferencing has been widely used in response to COVID-19 and has proven effective to obtain a quick evaluation on the evolution of COVID-19.

Finally, a dynamic perspective for the design of a flexible lockdown mechanism is required. Our study [5] finds the low effectiveness to mitigate the transmission of COVID-19 in both Japan and the USA has been caused by the low viral test rates at the earlier stages of the epidemic. Thus, a dynamic response system may provide rapid response by integrating all relevant information to formulate a prompt and effective response strategy.

## Figures and Tables

**Table 1:** The epidemiological data of some Latin American countries with the most confirmed cases in the world.

	New Cases in 17–23 August	Confirmed Cases by 23 August	PCP on 23 August (Per Million)	PNC	Ranking on 28 August
Argentina	46,606	329,043	7280	14.16%	11
Brazil	256,810	3,532,330	16,618	7.27%	2
Chile	11,806	395,708	20,700	2.98%	10
Columbia	77,027	522,138	10,262	14.75%	8
Mexico	38,365	549,734	4264	6.98%	7
Peru	59,771	576,067	17,471	10.38%	6

**Table 2:** The daily viral tests and positive rates for countries selected.

	Daily Tests Per 1000	Date of Daily Tests	Positive Rates	Date of Positive Rates
Argentina	0.25	20 August 2020	57.90%	20 August 2020
Brazil	0.43	22 August 2020	n.a.	n.a.
Chile	1.42	27 August 2020	6.50%	27 August 2020
Columbia	0.59	27 August 2020	33.40%	27 August 2020
Mexico	0.08	21 August 2020	52.40%	21 August 2020
Peru	0.26	18 August 2020	n.a.	n.a.
USA	2.24	25 August 2020	5.80%	25 August 2020
